# Relationship between Bone Health Biomarkers and Cardiovascular Risk in a General Adult Population

**DOI:** 10.3390/diseases5040024

**Published:** 2017-10-24

**Authors:** Cristina Vassalle, Laura Sabatino, Pietro Di Cecco, Maristella Maltinti, Rudina Ndreu, Silvia Maffei, Alessandro Pingitore

**Affiliations:** Fondazione CNR-Regione Toscana G Monasterio and Istituto di Fisiologia Clinica, CNR via Moruzzi 1, I-56124 Pisa, Italy; laura.sabatino@ifc.cnr.it (L.S.); pietro.dicecco@ftgm.it (P.D.C.); maristella@ftgm.it (M.M.); rudina.ndreu@ifc.cnr.it (R.N.); silvia.maffei@ftgm.it (S.M.); pingi@ifc.cnr.it (A.P.)

**Keywords:** cardiovascular risk, vitamin D, bone turnover biomarkers, FRAMINGHAM score, PROCAM score

## Abstract

Purpose/Introduction: Osteoporosis (OP) and cardiovascular (CV) disease emerge as closely related conditions, showing common risk factors and/or pathophysiological mechanisms. The aim of this study was to evaluate the association between bone health markers (BHM) and individual CV risk factors and overall CV risk (FRAMINGHAM-FRS, and PROCAM scores) in a general adult population. Methods: In 103 subjects (21 males; age: 56 ± 12 years), vitamin D (25(OH)D), osteocalcin (OC), bone alkaline phospatase (BALP), procollagen I aminoterminal propeptide (P1NP), CTx-telopeptide, as well clinical history and life style were evaluated. Results: Aging (*p* < 0.001) and glycemia (*p* < 0.05) emerged as independent 25(OH)D predictors. Aging (*p* < 0.001), male sex (*p* < 0.05), and obesity (*p* < 0.05) represented independent OC determinants. Aging (*p* < 0.05) was the only independent BALP determinant. After multivariate adjustment, low 25(OH)D (<20 ng/mL) (Odds ratio OR (95% confidence intervals CI)) (5 (1.4–18) *p* < 0.05) and elevated OC (>75th percentile-16.6 ng/mL) (6.7 (1.9–23.8) *p* < 0.01) were found to be significant FRS predictors, while subjects with elevated OC and/or BALP (>75th percentile-9.8 μg/L) showed a higher CV risk as estimated by PROCAM (3.6 (1.2–10.7) *p* < 0.05). CTx and P1NP did not significantly correlate with CV risk factors or scores. Conclusion: As we go further into bone and CV physiology, it is evident that a close relationship exists between these diseases. Further studies are needed to investigate mechanisms by which bone turnover markers are related to metabolic risk and could modulate CV risk. This knowledge may help to develop possible multiple-purpose strategies for both CV disease and OP prevention and treatment.

## 1. Introduction

Both osteoporosis (OP) and cardiovascular (CV) disease are relevant public health problems, leading to increased morbidity and mortality as well as elevated clinical and economic burden [1]. Recent data suggest the relationship between osteoporosis and CV disease through mechanisms that have not been fully elucidated, but are likely related to common risk factors, common pathophysiological mechanisms, or both [1]. Bone health markers (BHM) showed correlation with biomarkers of subclinical atherosclerotic manifestation, CV risk factors and events [1]. In particular, osteocalcin (OC), a bone matrix protein produced by osteoblasts, has been investigated as a hormone affecting glucose metabolism and fat mass [2]. Moreover, several evidences suggest the relationship between low 25-hydroxyvitamin D (25(OH)D) and different cardiovascular determinants, particularly aging, hypertension, diabetes, obesity, and metabolic syndrome [3,4,5]. Low 25(OH)D has been related to higher CV risk and CV and overall mortality through meta-analysis studies [6,7].

In recent years, different scales—including the PROCAM and the FRAMINGHAM (FRS)—have been developed to estimate the 5–10-year risk in asymptomatic population [8,9]. However, BHM have never been evaluated according to global risk. 

Thus, the aim of the present study is to evaluate the associations between biochemical BHM, individual CV risk factors, and CV risk scores in a general adult population.

## 2. Material and Methods

### 2.1. Subjects and Cardiovascular Risk Scores

The study included a general population of 103 subjects (21 males; mean age (SD): 56 (12) years) including adult volunteers and hospital outpatients of our Endocrinological Ambulatory in Pisa, Italy (latitude 43° N). At enrolment, each participant was interviewed about CV disease familiarity, clinical history, previous CV events, and lifestyle habits. Hypertension was defined if the average of recorded values was higher than 140/90 mmHg or in the presence of antihypertensive treatment. For each patient, body mass index (BMI) was calculated (obesity if BMI > 30 kg/m^2^). An altered lipid status was considered in the case of total cholesterol concentration ≥200 mg/dL (5.18 mmol/L), or triglyceride concentration ≥150 mg/dL (1.69 mmol/L), or current use of lipid-lowering drugs. Type 2 diabetes was defined if twice-fasting plasma glucose was >126 mg/dL (7 mmol/L) or in case of use of antidiabetic treatment. All subjects were free from acute or chronic inflammatory disease, significant renal impairment, immunological disease, and history or evidence of malignancy.

The FRS risk score predicts the 10-year risk of developing CV events, including coronary heart disease, stroke, peripheral artery disease, or heart failure. This sex-specific score considers age, total and high-density lipoprotein cholesterol, systolic blood pressure, treatment for hypertension, smoking, and diabetes. A score <10% is considered at low risk, 10–20% intermediate risk, and >20% high risk of CV events [8].

The PROCAM risk score considers age, LDL cholesterol, smoking, HDL cholesterol, systolic blood pressure, family history of premature myocardial infarction, diabetes mellitus, and triglycerides, in addition to previous coronary events [9]. A score lower than 10% is considered low, 10–20% intermediate, and higher than 20% as high for the 10-year risk of CV events.

We have complied with the World Medical Association Declaration of Helsinki regarding ethical conduct of research involving human subjects.

### 2.2. Laboratory Measurements

After an overnight fast, blood samples were drawn from the left antecubital vein, and centrifuged within 15 min after blood collection at 2500× *g* for 15 min at 4 °C. Serum samples were immediately stored at –80 °C for less than one week before subsequent analysis. 25(OH)D, OC, and bone alkaline phosphatase (BALP) were analyzed on an automated LIAISON (Diasorin, Salluggia, Vicenza, Italy), while procollagen type I N-terminal propeptide (P1NP) and C-terminal telopeptide (CTX) were also measured on a Cobas e411 analyzer (Roche Elecsys, Indianapolis, IN, USA) [3,10]. As we previously observed, 25(OH)D samples were extremely stable for light, temperature, and storage, without requiring special transport or precautions [3]. 25(OH)D was scored according to daylight saving time (DST), which is the is the practice of setting the clocks forward 1 hour from standard time during the summer months, and back again in the fall. Hemolyzed samples were excluded and withdrawal was repeated, as OC may decrease with hemolysis [10]. 

In addition, glycemia (fluoride-containing tubes), insulin (INS), lipid profile (total cholesterol, TotCH; triglycerides, TG; high density lipoproteins, HDL), and hsC-reactive protein (CRP, serum) were measured with a standard clinical chemistry laboratory analyzer (UniCel DxC 600 Chemistry Analyzer, Beckman, Brea, CA, USA). Levels of low-density lipoproteins (LDL) were calculated with the Friedewald equation.

### 2.3. Statistical Analysis

Continuous variables with little-to-mild skewness were summarized as means ± SD, while data were expressed as medians (min-max) for variables with a skewed distribution, or percentages for categorical variables. Comparisons were made by means of the two-sample Student’s *t*-test for continuous variables and by Chi-square analysis for categorical variables. Comparisons among different groups were performed by using ANOVA test and *p* for trend reported. Regression analysis with Pearson’s test was also used to evaluate the relationship between the two continuous variables.

Owing to skewness, log transformations of glycemia, INS, TG, OC, CTx, and CRP were used for statistical analyses. Then, log-transformed values were back-transformed for data presentation.

Univariate predictors with a *p* value ≤ 0.5 were entered into a multivariate regression analysis (BHM as the dependent variables, and significant parameters as the independent variables to determine independent predictors for each BHM). Additionally, a logistic regression analysis was used to estimate the independent association among BHM with the composite scores (FRS, PROCAM), after adjusting for potential confounders that were not part of the scores (CRP and obesity), but can increase CV risk and may be related to BHM.

A *p*-value < 0.05 was chosen as the level of significance.

## 3. Results

### 3.1. Characteristics of Subjects

Demographic and clinical characteristics of the subject population, lifestyle factors, and risk score levels are reported in Table 1. The studied population included 103 subjects (21 males; mean age (SD): 56 (12) years). The whole population included 11 obese subjects and 19 current smokers, as well as 48 subjects with dyslipidemia, 30 with hypertension, and 10 with Type 2 diabetes.

According to the baseline cardiovascular risk estimation, 86 subjects (84%) were classified as low risk, and 17 (16%) as intermediate risk with regard to FRS. Risk group distribution according to PROCAM was 85 (82%), 15 (15%), and 3 (3%) for the low-, intermediate-, and high-risk groups, respectively.

### 3.2. Correlation between Bone Health Biomarkers 

The correlation among BHM (OC, BALP; P1NP, CTx, 25(OH)D) is reported in Table 2. 

25(OH)D inversely correlated with OC (r = −0.30, *p* < 0.01) and BALP (r = −0.27, *p* < 0.01) (Table 2). All the other BHM directly correlated with each other. Specifically, OC directly correlated with BALP (r = 0.66, *p* < 0.001), P1NP (r = 0.53, *p* < 0.001), and CTx (r = 0.54, *p* < 0.001). Moreover, P1NP directly correlated with BALP (r = 0.45, *p* < 0.001), CTx with BALP (r = 0.45, *p* < 0.001), and P1NP with CTx (r = 0.82, *p* < 0.001) (Table 2).

### 3.3. Correlation between Bone Health Biomarkers and CV Risk Factors

#### 3.3.1. 25(OH)D

25(OH)D inversely correlated with glycemia (r = −0.32, *p* < 0.01) and aging (r = −0.47, *p* < 0.001). Moreover, 25(OH)D levels were higher in females (24 ± 9 versus 19 ± 11 ng/mL, *p* < 0.05), and lower in subjects with diabetes (14 ± 9 versus 24 ± 10 ng/mL, *p* < 0.01), hypertension (19 ± 8 versus 25 ± 10 ng/mL, *p* < 0.01), and dyslipidemia (21 ± 9 versus 26 ± 11 ng/mL, *p* < 0.05), with respect to those without (Table 3 and Table 4). Levels of 25-OHD were found to be sufficient (≥30 ng/mL) only in 21 (20%) subjects. 25(OH)D levels were higher during the DST period, although this difference did not reach statistical significance (25 ± 8 versus 22 ± 11 ng/mL, *p* = 0.2).

In a multiple regression model adjusted for the variables, aging (*t*-value −3.9, *p* < 0.001) and glycemia (*t*-value −2.2, *p* < 0.05) emerged as the only significant independent predictors for 25(OH)D.

**Figure 1:**
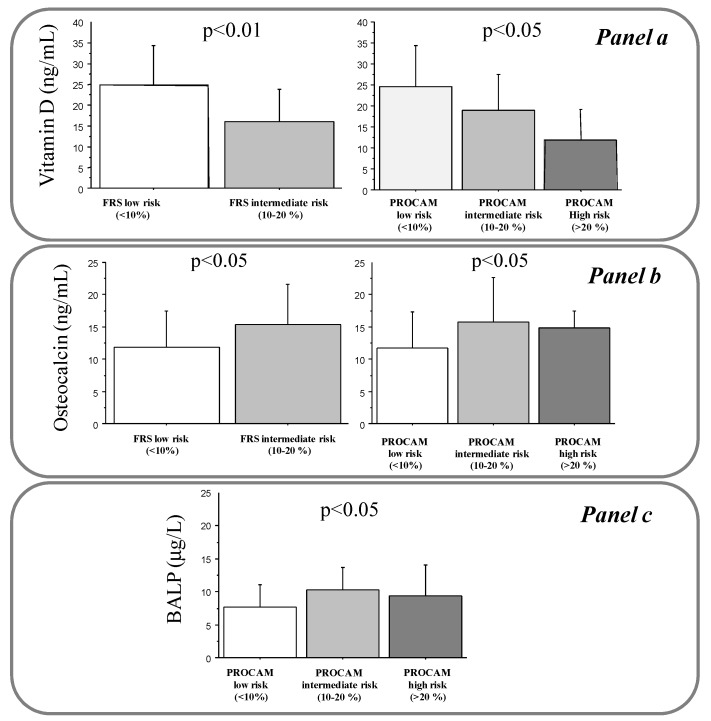
Levels of 25(OH)D (panel **a**), OC (panel **b**), and BALP (Panel **c**) according to FRS and PROCAM risk stratification in the studied population. *p* for trend.

Levels of 25(OH)D were also reduced in subjects with higher FRS (16 ± 8 versus 25 ± 10 ng/mL, *p* < 0.01) and PROCAM scores (12 ± 7 versus 19 ± 9 and 25 ± 10 ng/mL, *p* < 0.05 for trend) (Figure 1, panel a).

#### 3.3.2. OC

OC positively correlated with aging (r = 0.37, *p* < 0.001) and INS (r = 0.32, *p* < 0.01) (Table 3). Moreover, OC levels were higher in men than women (15 ± 5.8 versus 11.7 ± 5.8 ng/mL, *p* < 0.05), and lower in obese subjects (8.6 ± 3.3 versus 12.8 ± 6 ng/mL, *p* < 0.05), and during the DST period versus no-DST (11 ± 5 versus 13.3 ± 6.3 ng/mL, *p* ≤ 0.05) (Table 4). Multiple regression analysis revealed that aging (*t*-value 3.1, *p* < 0.001), male sex (*t*-value 2.4, *p* < 0.05), and obesity (*t*-value −2.5, *p* < 0.05) represented independent determinants for OC. However, OC levels increased according to increased FRS (11.8 ± 5.7 versus 15.4 ± 6.2 ng/mL, *p* < 0.05) and PROCAM risk stratification (11.7 ± 5.6 versus 15.8 ± 6.8 and 15 ± 2.6 ng/mL, *p* < 0.05 for trend) (Figure 1, panel b). 

#### 3.3.3. BALP

BALP directly correlated with aging (r = 0.3, *p* < 0.01) and INS (n = 65, r = 0.26, *p* < 0.05) (Table 3). Moreover, BALP levels were higher in dyslipidemic subjects (8.8 ± 3.8 versus 7.4 ± 3.2 μg/L, *p* ≤ 0.05) (Table 4). Aging (*t*-value 2.4, *p* < 0.05) remained the only significant determinant for OC at the multiple regression analysis. Only the PROCAM score was associated with BALP (9.3 ± 4.7 versus 10.3 ± 3.4 and 7.6 ± 3.4 μg/L, *p* < 0.05 for trend) (Figure 1, panel C).

#### 3.3.4. CTx and P1NP

CTx and P1NP levels were significantly lower during DST with respect to the no-DST period (0.24 ± 0.14 versus 0.32 ± 0.2 ng/mL, *p* ≤ 0.05; 27 ± 13.2 versus 37 ± 16 μg/L, *p* ≤ 0.01, respectively), without showing any other significant correlation with individual cardiovascular risk factors, neither with FRS nor PROCAM (Table 3 and Table 4).

#### 3.3.5. Logistic Analysis for FRS and PROCAM

For this analysis, OC and BALP were scored according to the 75th percentile (16.6 ng/mL and 9.8 μg/L, respectively), while 25(OH)D was scored using the cut-off of 20 ng/mL. After adjustments for obesity and CRP (scored according to the 75th percentile corresponding to 0.3 mg/dL), low 25(OH)D (OR(95%CI), 5 (1.4–18) *p* < 0.05) and elevated OC (6.7 (1.9–23.8) *p* < 0.01) levels were found to be significant predictors for FRS, while the finding of elevated OC and/or BAP remained independently associated with PROCAM (3.6 (1.2–10.7), *p* < 0.05).

## 4. Discussion

Our results suggest a significant relationship between 25(OH)D, OC, and BALP and major CV factors and global risk based on FRS and PROCAM scores in a general adult population.

With regard to individual CV risk factors, our data are in agreement with previous evidences. In particular, 25(OH)D, a recognized factor promoting bone health, retains many other “extraskeletal” actions [1]. In particular, biologically plausible mechanisms related to CV disease prevention have been identified, while observational studies also suggest an inverse association between serum 25(OH)D and CV risk [11]. In fact, 25(OH)D may modulate hepatic and pancreatic islet function affecting hepatic glucose and lipid metabolism, likely through the activation of Ca^2+^/CaMKK/AMPK signaling [12]. Moreover, it inhibits renin activity, suppresses the renin-angiotensin-aldosterone system, and affects nitric oxide levels, inflammatory parameters, angiogenesis, platelet aggregation, insulin resistance, and fasting glucose values [12,13,14,15]. Recently, another mechanism by which 25(OH)D exerts its beneficial effects has been identified in its capacity to promote antioxidant pathways and cope with oxidative stress and hyperglycemic damage [15]. Accordingly, a recent meta-analysis confirmed the inverse correlation of 25(OH)D with individual CV risk factors, including diabetes, hypertension, and dyslipidemia [16]. 

Generally, 25(OH)D levels vary across the course of the year (3). In the present study, although higher in DST, levels of 25(OH)D did not significantly differ. We enrolled a relatively low number of subjects, including a few subjects in the spring and summer, and as such seasonal variation or month comparison and further inference and explanation of the 25OHD variability, not being focus of the present study, are limited. Nonetheless, we observed a trend showing higher levels at the end of summer and lower levels in winter, which was in agreement with the trend we previously observed in healthy adults [3]. Moreover, as the majority of patients (78%) in this cohort presented 25(OH)D insufficiency or deficiency, the seasonal effect was thought to be minor.

OC, a product of osteoblasts, has attracted much attention as a hormone with close interactions with glucose homeostasis and fat, and it shows the capacity to stimulate islets and fat to secret insulin [2]. In a subgroup of patients, we found a direct correlation between OC and INS, in agreement with previous experimental results, which evidenced that OC can favor normal INS secretion by pancreatic β cells [2,17,18]. Many studies evidenced the relationship between low OC and diabetes and atherosclerosis, including subclinical atherosclerotic biomarkers [2]. Thus, the increase of OC with global risk scores found in our population may appear surprising, considering the inverse relationship of OC with diabetes and obesity indices. However, other authors have reported opposite results, including a higher prevalence of carotid atherosclerosis in healthy post-menopausal women with elevated OC and low BMD, or no correlation [2,19,20]. In this scenario, the complexity of the relationship between OC and CV disease is evidenced by results showing that elevated OC may also predict CV risk and mortality. In particular, increased OC was found to be involved in the pathogenesis of coronary artery disease in patients with chronic kidney disease. A large cohort study including elderly men (3542 men aged 70–89 years followed for a median of 5.2 years with 198 fatal CV disease events) found the highest CV disease mortality at a high serum OC concentration, as well as in a large cohort of patients at high CV risk (2271 men referred to coronary angiography) [21,22]. In this context, it is critical to consider the relationship with other biomarkers and age-related changes in bone turnover. In fact, we observed an inverse relationship between 25(OH)D with other BHM, with a positive correlation between the two. Moreover, aging represents an independent factor for 25(OH)D, OC, and BALP in our population. The risk induced by high OC levels could be mediated by arterial calcification, since OC is expressed in calcified atherosclerotic lesions, where it represents a mediator in the calcification process. Accordingly, OC levels strongly correlated with mitral annular calcification in patients with chronic kidney disease [23]. Interestingly, early, highly active endothelial progenitor cells (EPCs), carrying the osteoblastic marker OC, appear to be strongly associated with unstable coronary artery disease suggesting that this particular EPC subset could mediate vascular calcification and abnormal vascular repair and may identify patients with a more unstable phenotype of atherosclerosis [24]. Moreover, a higher percentage of circulating EPCs expressed OC in patients with coronary atherosclerosis compared to subjects with normal endothelial function [25].

There are few data on the relationship between BALP and CV risk. A significant association has been evidenced between BALP and arterial stiffness in pre-dialysis chronic kidney disease [26]. We found higher BALP levels in subjects with dyslipidemia. However, as the role of statins in the reduction of BALP levels has been previously evidenced, we verify the lack of any effect of statin treatment on BALP levels in our population (data not shown) [27]. Data for P1NP and CTx on CV risk, for which we did not find any relationship with CV risk, are even more scarce. Nonetheless, CTx together with OC levels were found to be lower in Type 1 diabetes children than in healthy controls [28]. Moreover, a negative correlation between CTx levels and glycated hemoglobin was observed in diabetic male subjects [29]. In addition, weight-loss has been associated with significant variation in bone formation as well as resorption biomarkers, including OC and CTx [30,31,32,33,34]. Nonetheless, a U-shaped association of CTx levels, together with OC, with CV death and overall mortality was observed in a large cohort of men at high CV risk, suggesting that there may exist an optimum, intermediate range which identifies the highest benefits for both biomarkers [21]. Instead, higher P1NP was associated with higher incidence of myocardial infarction (MI), but not of stroke, whereas CTx was not associated with incident MI or stroke [35]. Thus, the effects of these last biomarkers must be further assessed in other trials and in different populations of patients to assess their potential and significance in specific clinical settings.

Since the relatively low number of subjects enrolled may represent a limitation, a power analysis was performed to test the association between bone turnover biomarkers and CV risk. Thus, according to the method of Sheppard [36], we chose a medium effect size (i.e., a standardized difference) of 0.5, with an α value of 0.05. The program G*Power 3.1.2 was used for the calculation. The effective power of the study was determined to be 0.99.

## 5. Conclusions

As we go further into bone and CV physiology, it is evident that a close relationship exists between these conditions. Further studies are needed to investigate potential mechanisms by which bone turnover markers are related to metabolic risk and could modulate CV risk. From an holistic perspective, it is conceivable that patients affected by OP likely benefit from CV evaluation, whereas patients with CV disease would benefit from bone health assessment. Moreover, it will be also interesting to understand how common biological and molecular factors may drive the progression towards a certain pathology and explain why a subject will develop one specific disease rather than another.

## Figures and Tables

**Table 1:** Demographic, clinical and biochemical characteristics of the studied subjects.

Parameters	Values
n	103
Age (years)	56 ± 12
Males, n (%)	21 (20)
Hypertension, n (%)	30 (29)
Diabetes, n (%)	10 (10)
Dyslipidemia, n (%)	48 (47)
Current smokers, n (%)	19 (19)
Obesity, n (%)	11 (11)
Body Mass Index (Kg/m^2^)	24.8 ± 4.6
Total cholesterol (mg/dL)	207 ± 36
HDL cholesterol (mg/dL)	62 ± 16
Triglycerides (mg/dL), median (min-max)	89 (39–299)
LDL cholesterol (mg/dL)	125 ± 30
Glycemia (mg/dL), median (min-max)	88 (73–161)
Insulin (mUI/mL) *, median (min-max)	4 (1–18)
Systolic blood pressure (mmHg)	125 ± 18
Diastolic blood pressure (mmHg)	75 ± 9
C-reactive protein (mg/dL), median (min-max)	0.14 (0.02–3.1)
25-hydroxyvitamin D (ng/mL)	23 ± 10
Osteocalcin (ng/mL), median (min-max)	11 (5–32)
Bone alkaline phosphatase (mg/L)	8 ± 3.6
Amino-terminal propeptide (mg/L)	32.4 ± 15.5
C-terminal telopeptide (ng/mL), median (min-max)	0.2 (0.1–0.8)

* data available in 65 subjects. Values are mean ± DS, unless otherwise specified.

**Table 2:** Correlation between bone health biomarkers.

	OC	BALP	P1NP	CTx
25(OH)D	r = −0.3 *p* < 0.01	r = −0.27 *p* < 0.01	r = −0.1 *p* = ns	r = −0.15 *p* = ns
OC		r = 0.66 *p* < 0.001	r = 0.53 *p* < 0.001	r = 0.54 *p* < 0.001
BALP			r = 0.45 *p* < 0.001	r = 0.45 *p* < 0.001
P1NP				r = 0.82 *p* < 0.001

25(OH)D = vitamin D. OC = osteocalcin. BALP = bone alkaline phosphatase. P1NP = procollagen type I N-terminal propeptide. CTx = C-terminal telopeptide.

**Table 3:** Correlation between HBM and continuos variables.

	Age	BMI	Glycemia	Insulin *	Total Cholesterol	HDL	TG	LDL	CRP	SBP	DBP
	Years	Kg/m^2^	mg/dL	mUI/mL	mg/dL	mg/dL	mg/dL	mg/dL	mg/dL	mmHg	mmHg
VitD (ng/mL)	r = −0.47, *p* < 0.001	r = − 0.17, ns	r = −0.32, *p* <0.01	r = −0.07, ns	r = 0.02, ns	r = 0.2, ns	r = −0.16, ns	r = 0.01, ns	r = 0.13, ns	r = 0.1, ns	r = 0.03, ns
OC (ng/mL)	r = 0.37, *p* < 0.001	r = 0.02, ns	r = 0.18, ns	r = 0.32, *p* <0.01	r = 0.1, ns	r = −0.15, ns	r = 0.1, ns	r = 0.05, ns	r = 0.04, ns	r = 0.06, ns	r = 0.03, ns
BALP (mg/L)	r = 0.3, *p* < 0.01	r = 0.002, ns	r = 0.18, ns	r = 0.26, *p* <0.05	r = 0.13, ns	r = −0.13, ns	r = 0.02, ns	r = 0.17, ns	r = 0.07, ns	r = 0.18, ns	r = 0.1, ns
P1NP (mg/L)	r = 0.05, ns	r = 0.002, ns	r = −0.13, ns	r = −0.12, ns	r = 0.12, ns	r = 0.02, ns	r = 0.07, ns	r = 0.13, ns	r = 0.05, ns	r = −0.12, ns	r = 0.02, ns
CTx (ng/mL)	r = 0.1, ns	r = 0.01, ns	r = 0.03, ns	r = 0.08, ns	r = 0.2, ns	r = 0.01, ns	r = 0.03, ns	r = 0.17, ns	r = 0.1,ns	r = 0.01, ns	r = 0.09, ns

* data available in 65 subjects. ns = not significant.

**Table 4:** Correlation between HBM and categorical variables.

	Males	Diabetes	Hypertension	Dyslipidemia	Smoking Habit	Obesity	DST
VitD (ng/mL)	-*	-**	-**	-**	ns	ns	ns
OC (ng/mL)	+*	ns	ns	ns	ns	-*	-*
BALP (mg/L)	ns	ns	ns	+*	ns	ns	ns
P1NP (mg/L)	ns	ns	ns	ns	ns	ns	-**
CTx (ng/mL)	ns	ns	ns	ns	ns	ns	-*

* *p* ≤ 0.05, ** *p* ≤ 0.01.
